# Modulating the optical and electrical properties of MAPbBr_3_ single crystals via voltage regulation engineering and application in memristors

**DOI:** 10.1038/s41377-020-00349-w

**Published:** 2020-06-30

**Authors:** Jun Xing, Chen Zhao, Yuting Zou, Wenchi Kong, Zhi Yu, Yuwei Shan, Qingfeng Dong, Ding Zhou, Weili Yu, Chunlei Guo

**Affiliations:** 1 The Guo Photonics Laboratory, State Key Laboratory of Applied Optics, Changchun Institute of Optics, Fine Mechanics and Physics, Chinese Academy of Sciences, 130033 Changchun, China; 2 University of Chinese Academy of Sciences, 100049 Beijing, China; 3 State Key Laboratory of Supramolecular Structure and Materials, Jilin University, 130012 Changchun, China; 4 State Key Laboratory of Luminescence and Applications, Changchun Institute of Optics, Fine Mechanics and Physics, Chinese Academy of Sciences, 130033 Changchun, China; 5 The Institute of Optics, University of Rochester, Rochester, NY 14627 USA

**Keywords:** Photonic devices, Ultrafast photonics

## Abstract

Defect density is one of the most significant characteristics of perovskite single crystals (PSCs) that determines their optical and electrical properties, but few strategies are available to tune this property. Here, we demonstrate that voltage regulation is an efficient method to tune defect density, as well as the optical and electrical properties of PSCs. A three-step carrier transport model of MAPbBr_3_ PSCs is proposed to explore the defect regulation mechanism and carrier transport dynamics via an applied bias. Dynamic and steady-state photoluminescence measurements subsequently show that the surface defect density, average carrier lifetime, and photoluminescence intensity can be efficiently tuned by the applied bias. In particular, when the regulation voltage is 20 V (electrical poling intensity is 0.167 V μm^−1^), the surface defect density of MAPbBr_3_ PSCs is reduced by 24.27%, the carrier lifetime is prolonged by 32.04%, and the PL intensity is increased by 112.96%. Furthermore, a voltage-regulated MAPbBr_3_ PSC memristor device shows an adjustable multiresistance, weak ion migration effect and greatly enhanced device stability. Voltage regulation is a promising engineering technique for developing advanced perovskite optoelectronic devices.

## Perovskite devices: Digital memories thrive by tuning out defects

Innovative materials gaining favor as replacements for silicon solar cells can also be transformed into memory logic circuits using a new fabrication procedure. Lead halide perovskites can be turned into optoelectronic devices through low-cost solution depositions, but these approaches often leave numerous charge-trapping defects in the perovskite. Weili Yu from China’s Changchun Institute of Optics and colleagues have now developed a technique for modifying the defect population of perovskite crystals without requiring chemical additives. The team used probes to apply an electric field to the surface of a perovskite sample for helping move injected charges into defect sites with a high degree of control, which further modulated the optical and electrical properties of perovskite sample. Optimized defect populations enabled the perovskite to act as memristor device, capable of activating multiple resistance states.

## Introduction

Perovskite materials have been used in a variety of optoelectronic devices, such as solar cells^1–3^, photodetectors^4,5^, field effect transistors^6–8^, lasers^9,10^, and light emitting diodes^11,12^, due to their excellent intrinsic properties^13–15^. Continuously improving the performance of these optoelectronic devices is needed to overcome the bottleneck problem. The defect (including surface defects and volume defects) density in perovskites is a key parameter that limits the performance of these materials^16^. To control the surface defects, a widely studied method is to passivate and cure the defects by a surface engineering process, which can be achieved by adding a variety of additives, including ammonium methyl bromide^12^, guanidinium bromide^17^, potassium iodide^18^, phenethyl iodide^19^, poly(3-hexylthiophene-2,5-diyl)^20^, choline iodine^21^, and 1-butyl-3-methylimidazolium tetrafluoroborate^22^. However, this method requires precise control of the amount of the additives, the order of addition, and the reaction time, which makes this process complicated and results in a high risk of loss. To tune the volume defects, a known strategy is irradiating perovskite with high-energy ultraviolet light^23^, sunlight^24^, near-infrared light^25^, etc. This strategy requires a long repair time and sometimes results in irreversible damage to the materials, which makes the process complicated. Therefore, highly efficient and convenient pathways to regulate defects in perovskites are still needed.

Applying bias to perovskites has been reported to affect the fundamental properties of the perovskites under certain conditions. For example, the Huang group reported that piezoelectric poling could achieve grain polarization and ion migration in perovskite polycrystalline films, which introduced new defects when the electrical poling intensity was above 1.0 V μm^−1^^26,27^. Furthermore, at an electrical poling intensity larger than 0.5 V μm^−1^, the applied bias is thought to cause electroluminescence (EL) in perovskites^11,28^. However, despite the possibility of engineering perovskites, there are still bottlenecks in the advancement of perovskite applications, particularly in the case of perovskite single crystals (PSCs), which have been reported by Shi^14^ and Dong^15^ to have an ultralow trap density and a large carrier lifetime. For applications such as solar cells, lasers, LEDs or transistors, how the fundamental parameters can be tuned to maximize the device efficiency is still unclear, although it is critical. Studying defect regulation in PSCs under low electrical poling will be conducive to exploring novel ways to improve device performance based on PSCs. Aside from being convenient and easy to control, voltage regulation can be dynamically tracked by steady-state photoluminescence (PL) and time-resolved photoluminescence (TRPL) measurements in situ^29^, which provides specific insight into how the defect density in the MAPbBr_3_ single-crystal bulk (MPB SCBK) evolves.

Here, we first propose a three-step carrier transport model of MPB SCBK for the carrier transport mechanism under different applied biases. Then, both steady-state and dynamic PL measurements are conducted to explore the defect regulation and carrier transport dynamics under single-photon excitation (532 nm laser) in both the center region and regions around the cathode/anode. The best regulation result is achieved at 20 V, corresponding to an electrical poling intensity of 0.167 V μm^−^^1^. Finally, the first MPB SCBK memristor is fabricated, which shows tunable resistance and an ultrastable switching effect at each applied bias. The memristor overcomes the effect of ion migration and satisfies commercial application requirements. This research indicates that voltage regulation is an efficient technique for regulating defect density, carrier lifetime, PL intensity, and resistance.

## Results

### The mechanism of the three-step carrier transfer model of MPB SCBK with applied bias

**Fig. 1:**
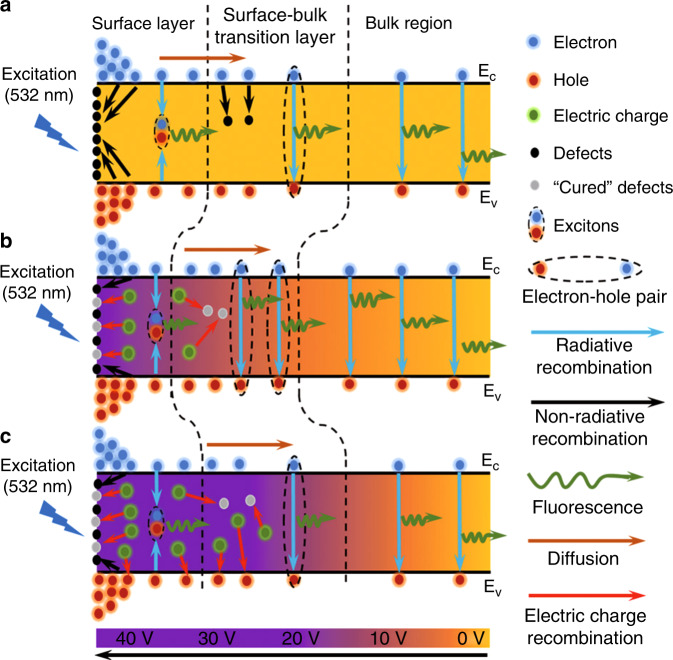
Schematic diagram of the three-step carrier transfer model for MPB SCBK with 532 nm excitation under different applied biases. **a** no bias, **b** appropriate bias and **c** excessive bias is applied

As shown in Fig. 1, we first propose a developed three-step carrier transport model to clarify the dynamic carrier transport mechanism of MPB SCBK based on previous reports^30–32^. The three steps, which occur at the surface layer, the surface–bulk transition layer and the bulk region, are the exciton recombination (short lifetime *τ*_1_), electron–hole pair recombination (middle lifetime *τ*_2_) and free-carrier recombination (long lifetime *τ*_3_) processes in TRPL measurements^32,33^. Upon excitation at 532 nm, electrons and holes are formed in the conduction band (*E*_c_) and valence band (*E*_v_), respectively, with a maximum concentration at the surface^31^. When no bias is applied, as shown in Fig. 1a, evaporation of the organic component leads to excessive lead halide at the MPB SCBK surface, which introduces more electron or hole defects at the surface^34–36^. Surface defects, mainly lead defects showing the ability to accept electrons such as a Lewis acid^36^, trap electrons via a non-radiative recombination mechanism at the surface. The electron–hole pairs, i.e., excitons, bounded by a Coulomb force produce fluorescence through exciton recombination in the surface layer^32^. The remaining carriers (electrons and holes) diffuse inside^37^. MPB SCBK under moderate growth conditions (the stoichiometric ratio of Br/Pb is 3:1) shows unavoidable volume defects, which are mainly deep-level donor-like point defects inside the MPB SCBK^38^. Some carriers become trapped under Shockley–Read–Hall (SRH) recombination during the diffusion process, whereas bounded electron–hole pairs in *E*_c_ and *E*_v_ emit fluorescence in the surface–bulk transition layer via electron–hole pair recombination^32^. Finally, after diffusion over a long distance, the unbounded free carriers with freely diffusive motion enter the bulk region and emit fluorescence with a long lifetime through free-carrier recombination^32,39^.

When the appropriate bias is applied, as shown in Fig. 1b, the injected charges can be trapped by lead defects in the surface layer^40,41^. The deep-level donor-like defects inside the bulk are much more sensitive to the applied bias because the bulk-charge recombination is sensitive to the total injected-charge density^36,42^. Furthermore, the deep-level donor-like defects are expected to reach the MPB SCBK surface under an electrical field^36,42^. The injected charges act as a Lewis base to passivate the deep-level donor-like defects inside the MPB SCBK^36^. These “cured” defects no longer trap carriers, and the probability of radiation recombination in the surface and surface–bulk transition layers is enhanced^43^. Furthermore, diffusion of the free carriers into the bulk region is induced, resulting in emission of more fluorescence through free-carrier recombination. Therefore, the passivation of surface defects through voltage regulation engineering could avoid trapped carriers and ultimately passivate the bulk defects of MPB SCBK as well^36^. The fractional contribution of the bulk region (*f*_3_) increases, while *f*_1_ (surface layer) and *f*_2_ (surface–bulk transition layer) are greatly reduced. This indicates that the surface layer and the surface–bulk transition layer attenuate their contribution to *τ*_ave_^44^. However, when excessive bias is applied, as shown in Fig. 1c, the excessive charges not only passivate the extended lead defects in the surface and deep-level point defects in the bulk but also trap holes through electric-charge recombination^36,45^. This means that the excessive injected charges act as new additive defects and reduce the probability of radiative recombination occurring. Thus, the carrier lifetimes and fractional contribution *f*_3_ decrease, while *f*_1_ and *f*_2_ increase. This indicates that the range of the surface layer and surface–bulk transition layer expands, leading to a decrease in *τ*_ave_^44^.

To prove the influence of applied bias on carrier transport, current-time characteristic curves of MPB SCBK under white light (36.4 mW cm^−^^2^) and in the dark are shown in Supplementary Figs. S1 and S2, respectively. Regardless of whether light or dark conditions are applied, the current output at different biases remains nearly constant for 60 s. The current–voltage (*J–V*) characteristic curves in Supplementary Figs. S1b and S2b are drawn from current-time characteristic curves. The *J–V* curves both show the same three-stage trend: the trap-filled region at a low voltage (<2 V), the charge injection region (the defects are passivated and cured in this region) at a medium voltage (2–20 V), and the charge-injected saturation region at a high voltage (>20 V)^14,15,46^. This indicates that injected charges influence carrier transport through voltage regulation. Detailed results and discussion are provided in the following sections.

### Experimental details and characterization

**Fig. 2:**
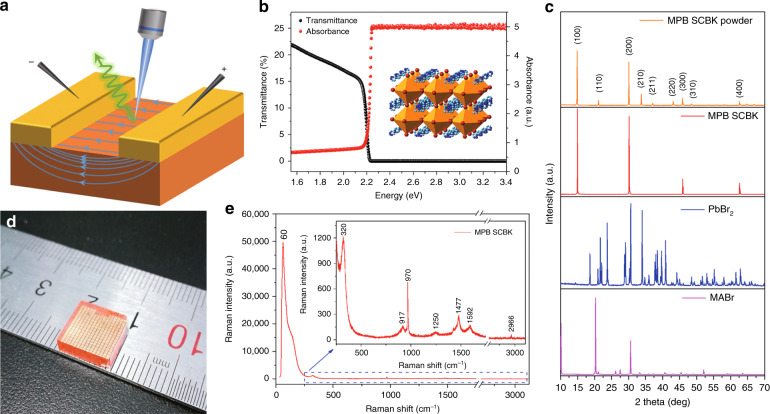
**Schematic of the experimental details and fundamental characterization.a** Schematic of the experimental device. The two probes are in contact with the gold electrode to provide a bias voltage, and the laser excites the MPB SCBK to detect dynamic PL and steady-state PL. **b** Absorption spectrum and transmission spectrum of MPB SCBK. The illustration shows the cubic structure of MPB SCBK. **c** XRD results of MPB SCBK powder, MPB SCBK, PbBr_2_, and MABr. **d** Optical image of centimeter size MPB SCBK with deposited gold electrode. **e** Raman spectrum of MPB SCBK

To verify our hypothesis, the experimental device is fabricated, and its schematic diagram is shown in Fig. 2a. The MPB SCBK with 100 nm gold electrodes is in contact with two probes connected to a DC power supply. The laser excites three regions between two electrodes of the MPB SCBK; the steady-state PL and TRPL that occur after applied bias regulation are then detected. The three regions include the region around the cathode (5 μm apart), the center region and the region around the anode (5 μm apart). A scanning electron microscopy (SEM) image and an energy dispersive spectrometry (EDS) map of MPB SCBK with a gold electrode are shown in Supplementary Fig. S3. The SEM image shows a clear boundary between the uniform MPB SCBK and the continuous gold electrode. The gold, bromine, lead, carbon, and nitrogen in the EDS map show even distributions and characteristic peaks in Supplementary Fig. 3g. Supplementary Figure S4 shows the optical image, three-dimensional (3D) pseudocolour plots and thickness information of the experimental device. The gold electrodes are 100 nm thick, continuous and arranged in parallel with an electrode spacing of 120 μm.

In Fig. 2b, the absorption spectrum and transmission spectrum of MPB SCBK show an absorption cut-off wavelength of ~560 nm. The absorption spectrum was measured in “Abs” mode without using the integration sphere. A thicker MPB SCBK (~3 mm) shows absorption supersaturation in the high-energy region. The X-ray diffraction (XRD) results of MPB SCBK powder, MPB SCBK, PbBr_2_ and MABr are shown in Fig. 2c. From the XRD results, it can be concluded that the two original materials (PbBr_2_ and MABr) have been completely transformed into MPB SCBK. The characteristic peaks in the MPB SCBK and MPB SCBK powder are consistent with cubic lattice structures (as shown in the inset of Fig. 2b). Figure 2d shows an optical image of the centimeter-sized MPB SCBK with a gold electrode. The MPB SCBK is grown from a 1.5 M mixed DMF solution of PbBr_2_ and MABr (molar ratio of 1:1) using the ITC method^47,48^ described in our previous report^49^. The Raman spectrum of MPB SCBK exhibits molecular vibrational peaks (60, 320, 917, 970, 1250, 1477, 1592, and 2966 cm^−1^) consistent with those in previous reports^50^, as shown in Fig. 2e. The characterizations demonstrate the high quality of the experimental device, which is further utilized for voltage-dependent experiments.

### TRPL measurement of MPB SCBK with an applied bias

The electrical poling intensity under various applied biases (0–50 V) is shown in Supplementary Fig. S5. Before measuring the TRPL, steady-state PL and *J*–*V* characteristics, the MPB SCBK is first polarized for 1 min under different biases. TRPL measurements are performed under 532 nm laser excitation, and the lifetime decay in three regions (around the cathode, in the center region and around the anode) is shown in Supplementary Figs. S6–S8, respectively. Through tri-exponential decay function fitting these lifetime decay curves, that is, short lifetime (*τ*_1_), middle lifetime (*τ*_2_), and long lifetime (*τ*_3_), and the corresponding fractional contributions (*f*_1_, *f*_2_, *f*_3_) can be obtained, as shown in each TRPL result. *τ*_ave_ can be calculated from $\tau _{ave} = \tau _1f_1 + \tau _2f_2 + \tau _3f_3$^51^. Detailed information is shown in Supplementary Tables S1–S3.

**Fig. 3:**
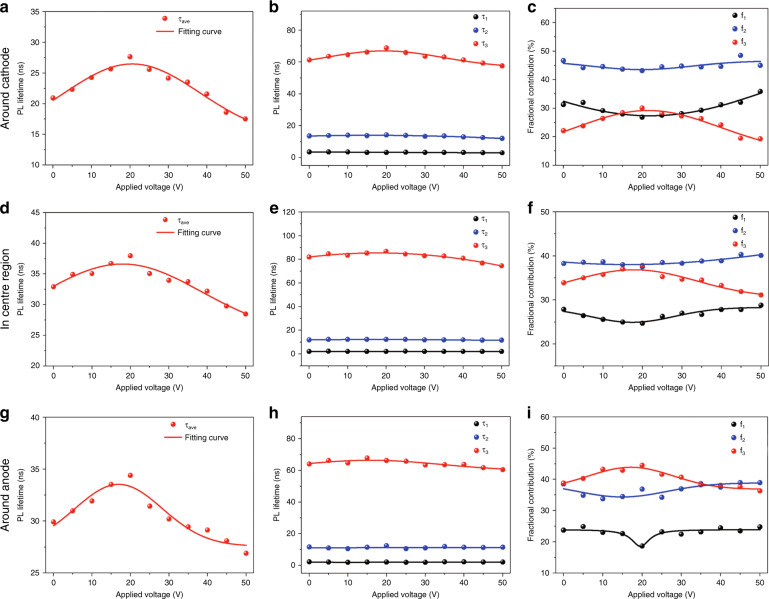
**Time-resolved photoluminescence (TRPL) characterization of MPB SCBK at different regions.a**–**c** Around the cathode, **d**–**f** in the center region and **g**, **h** around the anode with different applied biases. The variation in the **a**, **d**, and **g** PL average lifetime; **b**, **e**, **h** three-decay-process lifetimes (*τ*_1_, *τ*_2_, *τ*_3_), and **c**, **f**, **i** fractional contributions in the three-decay processes (f_1_, *f*_2_, *f*_3_) with different applied biases

The dependence of the carrier lifetime and fractional contribution on bias is shown in Fig. 3. In Fig. 3a, d, g, regardless of whether measured in the center region or around the cathode or anode, plots of *τ*_ave_ vs. applied bias exhibit Gaussian distributions. As the applied bias increases from 0 to 20 V, *τ*_ave_ shows an increasing trend and reaches a maximum at 20 V (the electrical poling intensity is 0.167 V μm^−^^1^). Then, it begins to decrease as the bias continues to increase. The champion *τ*_ave_ regulation results at 20 V (the electrical poling intensity is 0.167 V μm^−1^) increase by 32.04% around the cathode, by 11.47% in the center region and by 15.00% around the anode compared with the values with no bias regulation. After the bias is removed for 12 min, *τ*_ave_ is restored and becomes even larger than its initial value, as shown in Supplementary Tables S1–S3. This illustrates that applying bias is an effective technique to achieve the light emitting property, and the champion regulated bias is 20 V (the electrical poling intensity is 0.167 V μm^−1^). In addition, once the bias is removed, the MPB SCBK exhibits a certain degree of defect passivation, implying that voltage regulation can cure some defects and is thus a recoverable method.

The dependence of carrier lifetime components (*τ*_1_, *τ*_2_, and *τ*_3_) on applied voltage is summarized in Fig. 3b, e, h. *τ*_3_ shows an obvious voltage regulation effect, which follows the same trend as *τ*_ave_ in the three regions. This indicates that as the applied bias increases from 0 to 20 V, the injected charges passivate and cure the defects, thereby facilitating carrier transport and prolonging the free-carrier lifetime (*τ*_3_). When the applied bias is larger than 20 V, the injected charges are oversaturated and act as new defects to trap carriers. The fractional contributions (*f*_1_, *f*_2_, *f*_3_) in different regions also show a dependency on bias, as shown in Fig. 3c, f, i. *f*_1_ and *f*_2_ decrease first and then increase with increasing bias and exhibit a minimum value at ~20 V (the electrical poling intensity is 0.167 V μm^−^^1^). In contrast, *f*_3_ shows a Gaussian distribution that is consistent with the carrier lifetime (*τ*_3_ and *τ*_ave_). This further implies that charge injection has a regulatory effect on radiative recombination in each step to some extent. It is manifested as follows: under the same optical penetration depth^31^, as the applied bias increases to 20 V, appropriate charge injection not only plays a role in curing defects but also causes the surface and surface–bulk transition layers to shrink, while the bulk region range slowly increases. When the applied bias is >20 V, the excessive injected charges show the opposite effect on the three-layer region. The trend of carrier lifetime and fractional contributions further proves the three-step carrier transfer model with voltage regulation, as shown in Fig. 1.

**Fig. 4:**
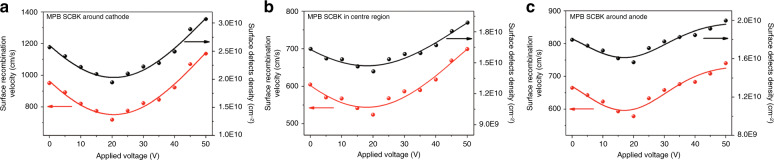
**The variation in surface recombination velocity and surface defect density in MPB SCBK at different regions.a** Around the cathode, **b** in the center region and **c** around the anode with different applied biases

Furthermore, according to the following equations^41,52^:

1 $$1/\tau _{\mathrm{S}} = \alpha S/\sqrt 2$$

2 $$S = {\upsigma}\nu _{{\mathrm{th}}}N_t$$

where *τ*_S_ is the lifetime under single-photon absorption (corresponding to *τ*_ave_)^41^, *α* is the absorption coefficient of ~70,862 cm^−1^^53^, *S* is the surface recombination velocity, *σ* is a typical recombination surface cross section in semiconductors (≈10^−15^ cm^−^^2^), *ν*_th_ is the carrier thermal velocity (≈3.7 × 10^7^ cm s^−1^), and *N*_*t*_ is the surface defect density. The simplified derivation process of Eq. 1 is detailed in the Supplementary Information. Whether in the center region or around the cathode and anode, as shown in Fig. 4, the calculated *S* and *N*_*t*_ values show the same trace as the surface layer fractional contribution (*f*_1_) and reach a minimum value at 20 V (the electrical poling intensity is 0.167 V μm^−1^), which further confirms the variation in the surface layer region and the defect-tuning effect. It is worth mentioning that the center region has lower *S* and *N*_*t*_ values than the regions near the electrodes. The champion defect regulation results at 20 V (the electrical poling intensity is 0.167 V μm^−^^1^) show a 24.27% reduction around the cathode, a 13.28% reduction in the center region and a 13.05% reduction around the anode compared with the values at no bias. These results imply the universality of the defect-tunable effect by voltage regulation engineering.

### Steady-state PL measurement of MPB SCBK with an applied bias

The steady-state PL intensity and carrier lifetime show a positive correlation as follows^51^:

3 $$I_{{\mathrm{SS}}} = {\int \nolimits_0^\infty} {I_0e^{ - t/\tau }{\mathrm{d}}t = I_0\tau }$$

where *I*_SS_ is the steady-state PL intensity, *I*_0_ is a parameter that depends on the fluorophore concentration and instrumental parameters, and *τ* is the average lifetime of the materials. Since the experimental conditions are fixed, the influence of *I*_0_ is ignorable, so *I*_SS_ is positively correlated with *τ*_ave_.

**Fig. 5:**
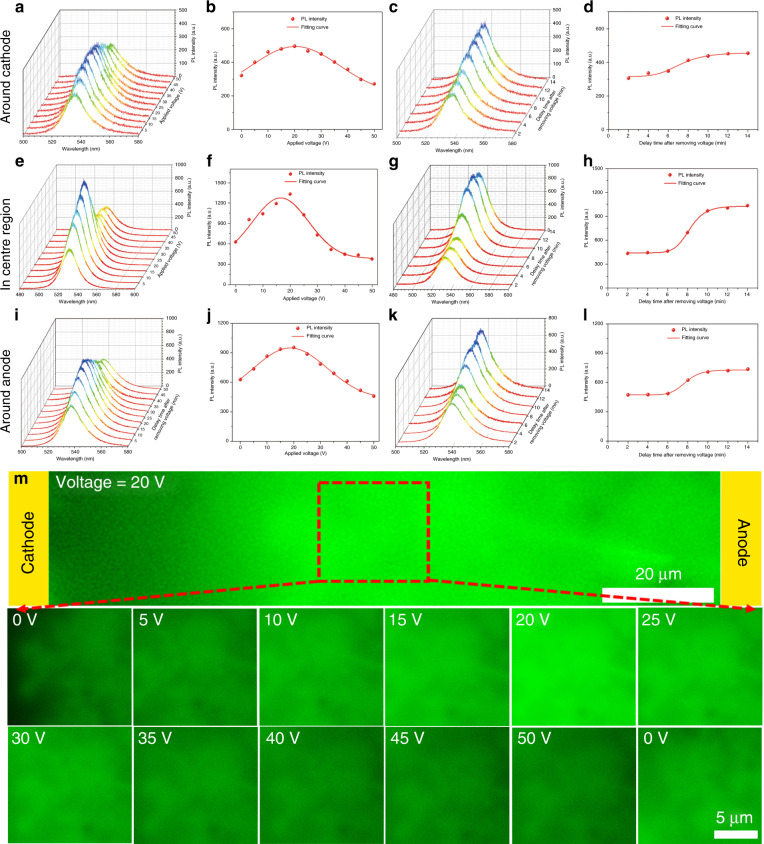
**Steady-state PL characterization of MPB SCBK at different regions.a**–**d** Around cathode, **e**–**h** in center region, and **i**–**l** around anode with different applied bias and removing bias. The variation in the **a**, **e**, and **f** waterfall map of the steady-state PL spectra and **b**, **f**, **j** PL maximum intensity with different applied biases. The variation in the **c**, **g**, and **k** waterfall map of the steady-state PL spectra and **d**, **h**, **l** PL maximum intensity after removing bias. **m** Fluorescence confocal micrograph of the area between the electrodes and the center region with different applied biases and upon removing the bias

The steady-state PL measurement is performed upon excitation with a 473 nm laser at room temperature, and the results are shown in Fig. 5. From a waterfall mapping of the steady-state PL spectrum in Fig. 5a, e, i, it can be concluded that there is no obvious peak shift, and the PL intensity shows a trend of increasing first and then decreasing as the applied bias increases. Figure 5b, f, j shows the variation in PL maximum intensity with increasing bias. The PL intensity shows the same trend as *τ*_ave_ and reaches a maximum value at 20 V (the electrical poling intensity is 0.167 V μm^−1^). The best PL intensity regulation results at 20 V (the electrical poling intensity is 0.167 V μm^−^^1^) show a 54.04% increase around the cathode, a 112.96% increase in the center region and a 51.83% increase around the anode compared with the values obtained with no bias regulation. Figure 5c, g, k shows the steady-state PL spectrum in different regions with time delay after removing bias. All PL intensities remain constant within 6 min, show a recovery process with a gradual increase between 6 and 10 min, and then stabilize after 12 min. Figure 5d, h, l shows the variation in PL maximum intensity with time delay. It is worth mentioning that the stable PL maximum intensity after 12 min is higher than the initial value, indicating that an appropriate charge injection can cure some defects and achieve more radiative recombination. To eliminate the effect of laser accumulation on PL intensity, the PL vs. time curve obtained at 0 V bias is shown in Supplementary Fig. S9. The results show a stable distribution of PL intensity over time, indicating that the above PL tunable results are affected by voltage regulation.

A more intuitive fluorescence confocal photomicrograph of MPB SCBK with different biases is shown in Fig. 5m. The fluorescence intensity in the center region is significantly higher than that around the electrodes at a bias of 20 V. The center region is chosen for detailed characterization. When 0 V bias is applied, uneven green fluorescence can be seen, and there is a certain region of weak fluorescence (black region). As the applied bias increases from 5 to 20 V, the weakly fluorescent region gradually disappears, and the overall fluorescence shows an increase, reaching its maximum at 20 V (the electrical poling intensity is 0.167 V μm^−^^1^). When the applied bias continues to increase, the fluorescence exhibits a decreasing trend. After bias is removed for 12 min, fluorescence in the last image is restored and is superior to the initial fluorescence, which is consistent with the PL intensity evolution shown above. The related parameters of the MPB SCBK in different positions before and after voltage regulation are summarized in Supplementary Table S4. The results show that after bias is removed, the PL intensity is enhanced, indicating that voltage regulation is a novel strategy to effectively regulate and cure defects. Therefore, the steady-state PL measurement results show the same voltage regulation effect as the TRPL measurements, again proving the universality of the three-step carrier transfer model for MPB SCBK and the defect-tunable effect by voltage regulation engineering.

### Memristor characteristics of MPB SCBK with an applied bias

**Fig. 6:**
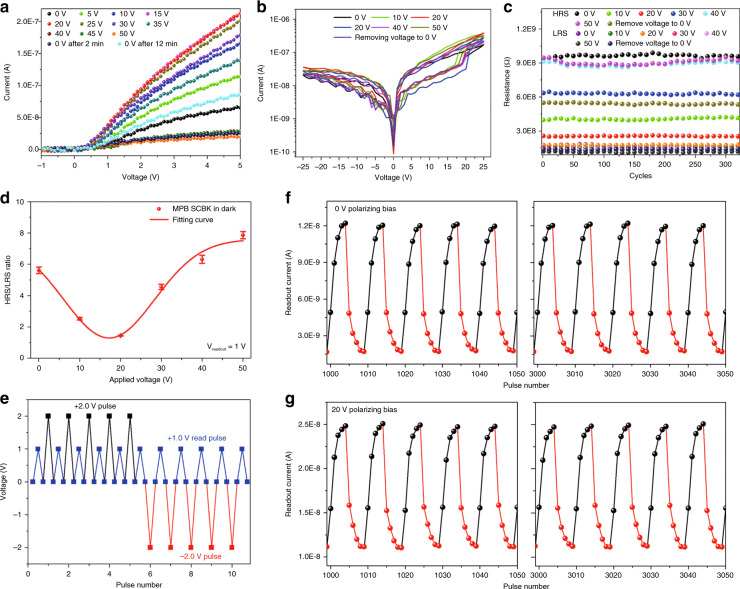
**Memristor characteristics of the Au/MPB SCBK/Au device**. **a***J–V* characteristic curves. **b***J–V* hysteresis loops. The voltage sweep sequence is 0 V → 25 V → 0 V → −25 V → 0 V. **c** Multiresistance states (HRS and LRS) at *V*_readout_ = 1 V. **d** The HRS/LRS ratio under different biases. The device was measured in the dark after different bias polarizations for 1 min. **e** Voltage trains applied on the device. The +2.0 and −2.0 V pulses (poling pulse duration: 0.1 s) were applied on the device, where the readout current was at +1.0 V (read pulse duration: 0.1 s). The time interval between the poling pulse and the read pulse is 0.1 s. **f**, **g** Readout currents of the device after 0 and 20 V polarizing bias, respectively

We further expand the application of voltage regulation in the first MPB SCBK memristor. In many perovskite polycrystalline thin-film memristors, ionic migration has been proposed as one of the mechanisms explaining the *J*–V hysteresis loops^54^. However, in our experiment, due to the small electrical poling intensity (<0.42 V μm^−^^1^) and the large ion migration energy in single-crystal materials, it is very difficult to induce ionic migration in MPB SCBK^55,56^. Thus, the MPB SCBK memristor (Au/MPB SCBK/Au structure device) is mainly attributed to charge trapping/detrapping mechanisms. Figure 6a shows the *J*–V characteristic curves of the device in the dark after polarization for 1 min under different applied biases. The turn-on voltage is always maintained at ~0.4 V without significant shifting, which rules out the effects of ionic migration^56^. Figure 6b shows the typical *J*–V hysteresis loops of the device under a voltage sweep sequence of 0 V → 25 V → 0 V → −25 V → 0 V. It can be concluded that different applied biases affect and regulate the *J*–*V* hysteresis loop curve of the memristor. As shown in Supplementary Fig. S10, voltage regulation yields a stable regulation result of the *J*–V hysteresis loop curve with more than 320 cycles. At a reading voltage of 1 V, as shown in Fig. 6c, it is worth noting that the regulation of different biases makes the Au/MPB SCBK/Au device exhibit multiresistance states with almost stable low resistance (LRS) and tunable high resistance (HRS). As the polarizing bias increases from 0 to 20 V, the HRS value shows a decreasing trend, which is mainly due to the defect passivation effect caused by injected charges. Conversely, when the polarizing bias continues to increase to 50 V, the HRS value exhibits an increasing tendency, which is mainly due to excess injected charges serving as new defects. The above changes are consistent with the charge trapping/detrapping mechanism^54^.

Detailed information on the device’s multiresistance under different poling biases is shown in Supplementary Fig. S11. Furthermore, the HRS/LRS ratios of the device under different polarizing biases at *V*_readout_ = 1 V are shown in Fig. 6d. The HRS/LRS ratio shows a change in the range from 1.44 to 8.1 under voltage regulation, which basically meets commercial application conditions. Although the HRS/LRS ratio of this device is lower than reported perovskite polycrystalline thin-film memristor^54,57^, which may be attributed to the low trap density of MPB SCBK and the negligible ionic migration effect, it still proves a high stability as a potential application in memristors. In addition, after removing the poling voltage for 12 min, the device shows an improvement in the HRS value, as shown in Supplementary Table S5, which further confirms that the curing effect of injected charges on defects is consistent with the measurement results in TRPL and steady-state PL.

For the memristor device, the operation speed, showing how fast the device can be switched between different states, is also an important criterion^54,58^. Figure 6e shows the applied poling pulse at +2.0 and −2.0 V with a 0.1 s poling pulse duration and a read pulse at +1.0 V, with a 0.1 s read pulse duration. As shown in Fig. 6f, g, the readout currents of the device under multiple voltage pulses after polarizing biases of 0 and 20 V show the same response speed of approximately a five-pulse switching time (0.5 s) between the LRS and HRS states. The fast switching time (0.5 s) in our MPB SCBK memristor compared with that of a perovskite polycrystalline thin film^57,59^ is mainly due to the charge trapping/detrapping mechanism^54^. In addition, the conductivity of the MPB SCBK memristor can be repeatedly tuned for more than 320 cycles with small fluctuations. Thus, voltage regulation engineering does not significantly change the response speed of the MPB SCBK memristor but only regulates the readout current value and affects the resistance of the device, which is consistent with the *J*–V hysteresis loops in Fig. 6b.

## Discussion

In summary, we demonstrate a three-step carrier transport model of MPB SCBK and voltage regulation engineering as an efficient strategy to regulate defects and influence dynamic carrier transport. The best voltage for regulation is achieved at 20 V (the electrical poling intensity is 0.167 V μm^−1^), wherein the average carrier lifetime is increased by 32.04%, the surface defect density is reduced by 24.27% and the PL intensity is increased by 112.96% compared with the values obtained with no bias. After removing the applied bias for 12 min, *τ*_ave_ and PL intensity are higher than their initial values, which indicates that a suitable voltage regulation (electrical poling intensity less than 0.42 V μm^−1^) will cure some defects in the MPB SCBK. Furthermore, voltage regulation shows a potential application on the first multiresistance adjustable (HRS/LRS ratio changing in range from 1.44 to 8.1) and ultrastable (more than 320 cycles) MPB SCBK memristor, which overcomes the effect of ion migration. This work provides novel insight into the flexibility of the defect density of perovskite SCBKs, and voltage regulation is an effective engineering method to tune not only the defect density but also the carrier lifetime, PL intensity, and resistance. This work will improve the optimization of optoelectronic devices based on PSCs.

## Materials and methods

### Materials

Methylamine solution (40% aqueous solution, Aladdin), lead bromide (PbBr_2_) (99%, Aladdin), and *N*,*N*-dimethylformamide (DMF) (99.5%, Aladdin) were purchased from Aladdin. Hydrobromic acid (HBr) (40% aqueous solution), absolute ethanol, and diethyl ether were purchased from Sinopharm Chemical Reagent Co., Ltd. All materials were used without further purification.

### Synthesis of methylammonium bromide (MABr)

MABr was synthesized according to our previous report. First, the two raw materials (44 mL HBr acid solution and 30 mL methylamine solution) were mixed in an ice bath for 2 h with stirring. Then, the white powder was recovered by rotary evaporation at 60 °C to remove the solvent. Next, the recovered white powder (MABr) was recrystallized by absolute ethanol and diethyl ether in turn three times. Finally, the recrystallized MABr was dried at 60 °C in a vacuum for one night.

### Synthesis of MAPbBr_3_ single-crystal bulk (MPB SCBK)

MPB SCBK was grown through an inverse temperature crystallization (ITC) method according to our previous report. First, 1.5 M MAPbBr_3_ precursor solution was prepared by mixing PbBr_2_ (1.5 mmol) and MABr (1.5 mmol) in DMF (10 mL) completely at room temperature. Then, a clear MAPbBr_3_ precursor solution (using a PTFE filter with 0.22 μm) was undisturbed and kept in an oil bath. Next, the oil temperature was gradually raised from room temperature to 75 °C. Finally, MPB SCBK with centimeter size was obtained after 3 h.

### Experimental device

The 100 nm gold electrode was evaporated on the surface of the MPB SCBK-covered template by a high vacuum metal evaporation coating system (ZHD-400, Beijing Technol Science Co., Ltd.). The electrode spacing was 120 μm. Different biases on the gold electrode were applied through two probes connected with a DC power supply (MCH-K605DN) to measure the steady-state PL and dynamic PL of the MPB SCBK.

### Time-resolved photoluminescence (TRPL)

The TRPLs of MPB SCBKs with different applied biases were measured by means of a home-built confocal microscope. A pulsed supercontinuum laser (OYSL Photonics, SC-Pro, 150 ps pulse lengths) at a 2 MHz repetition rate was used as the laser source. The focused pump laser (the wavelength was 532 nm after laser lines filter) power through an objective lens N.A. = 0.4 was 0.132 μW. A long-pass filter with a 532 nm edge (Semrock) was used to filter out the pump scattered light from the pump laser to the detector. The photoluminescence from MPB SCBK was detected by a SPCM-AQRH single-photon counting module (SPCM-AQRH-15, Excelitas Technologies), and the lifetime module was TimeHarp 260P (PicoQuant).

### Characterization of MAPbBr_3_ single-crystal bulk (MPB SCBK)

Current-time characteristic measurements of MPB SCBK were performed by using a Keithley 4200A semiconductor parametric analyser (Tektronix) and a C-100 probe station from TPSi Company in the dark at room temperature. The SEM image and energy dispersive spectrometry (EDS) mapping results of the gold electrode and MPB SCBK were measured by means of a Phenom Pro-X. A three-dimensional (3D) pseudocolour plot of the gold electrode deposited on the MPB SCBK was obtained by using a KEYENCE VK-X200 3D laser scanning microscope. The absorption spectrum and transmission spectrum were recorded on an Agilent Cary 5000. The XRD measurement was performed by using a BRUKER D8 FOCUS. Raman spectra were recorded on a HORIBA Scientific Raman spectrometer with 785 nm laser excitation in air at room temperature. Steady-state PL spectra of MPB SCBK with different applied voltages were obtained by means of a HORIBA Scientific Raman Spectrometer at 473 nm laser with 2.55 m W cm^−2^ laser intensity in air at room temperature. The PL confocal micrographs of MPB SCBK were obtained by Nikon ECLIPSE Ti with 486 nm laser excitation in air at room temperature. The *J–V* characteristic curves of MPB SCBK were obtained by using a Keithley 4200A semiconductor parametric analyser (Tektronix) and a C-100 probe station from TPSi Company in air at room temperature.

## Supplementary Information

Supplementary Information
