# Supplementary material for: Modulating the optical and electrical properties of MAPbBr3 single crystals via voltage regulation engineering and application in memristors
Source: Light Sci Appl. 2020 Jun 30;9:111. doi: 10.1038/s41377-020-00349-w (PMC7327067; doi:10.1038/s41377-020-00349-w)
Supplement: Supplementary file 1 — Supplementary Information [file 41377_2020_349_MOESM1_ESM.docx]

**Supplementary Information for**

**Modulating the Optical and Electrical Properties of MAPbBr_3_ Single Crystals via Voltage Regulation Engineering and Application in Memristors**

Jun Xing^1, 2,^ Chen Zhao^1, 2^, Yuting Zou^1, 2^, Wenchi Kong^1, 2^, Zhi Yu^1,2^, Yuwei Shan^1,2^, Qingfeng Dong^3^*, Ding Zhou^4^, Weili Yu^1,2^*, Chunlei Guo^1, 5^*

Correspondence: Weili Yu ([weili.yu@ciomp.ac.cn](mailto:weili.yu@ciomp.ac.cn)), Qingfeng Dong ([qfdong@jlu.edu.cn](mailto:qfdong@jlu.edu.cn)) or Chunlei Guo ([guo@optics.rochester.edu](mailto:guo@optics.rochester.edu))

^1^The Guo China-US Photonics Laboratory, State Key Laboratory of Applied Optics, Changchun Institute of Optics, Fine Mechanics and Physics, Chinese Academy of Sciences, Changchun 130033, China

^2^University of Chinese Academy of Sciences

Beijing 100049, China

^3^State Key Laboratory of Supramolecular Structure and Materials, Jilin University, Changchun, 130012, China

^4^State Key Laboratory of Luminescence and Applications, Changchun Institute of Optics, Fine Mechanics and Physics, Chinese Academy of Sciences, Changchun 130033, China

^5^The Institute of Optics, University of Rochester, NY 14627, USA

**Figure S1. Current-voltage characteristics of MPB SCBK under white light (36.4 mW cm^-2^) with different applied bias. a** Current-Time characteristic curves and **b** current-Voltage (*J*-V) characteristic curve of MPB SCBK under white light with 36.4 mW cm^-2^. The I-V curve shows a three-stage trend: the filling trap phase at low voltage (less than 2 V), the charge injection phase at medium voltage (2 V to 20 V), and the injected charge saturation phase at high voltage (greater than 20 V).


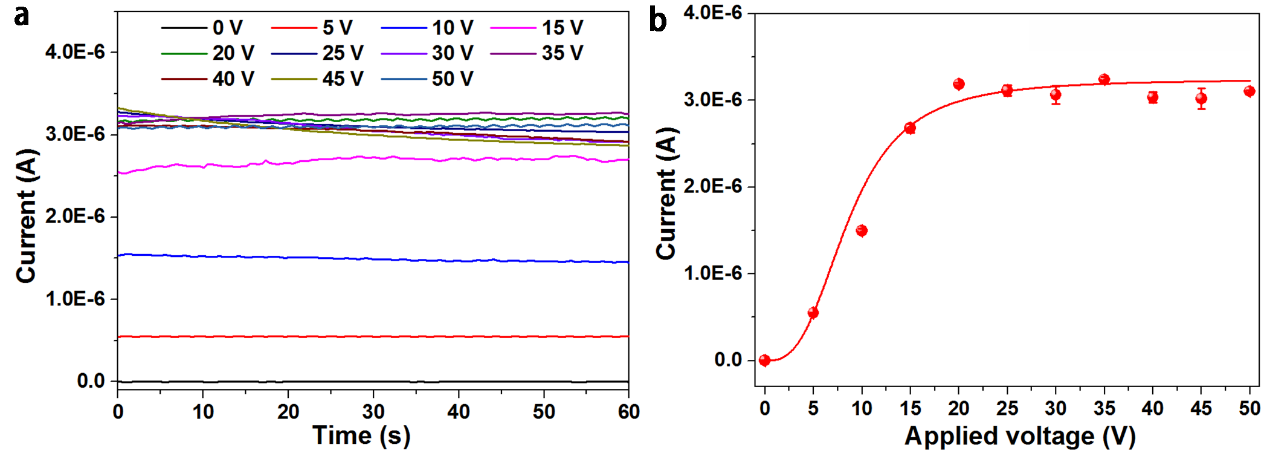


**Figure S2. Current-voltage characteristics of MPB SCBK in the dark with different applied bias. a** Current-Time characteristic curves and **b** current-Voltage (*J*-V) characteristic curve of MPB SCBK in the dark. The *J*-V curve in the dark shows same three-stage trend as MPB SCBK under white light with 36.4 mW cm^-2^ in supplementary Fig. S1.


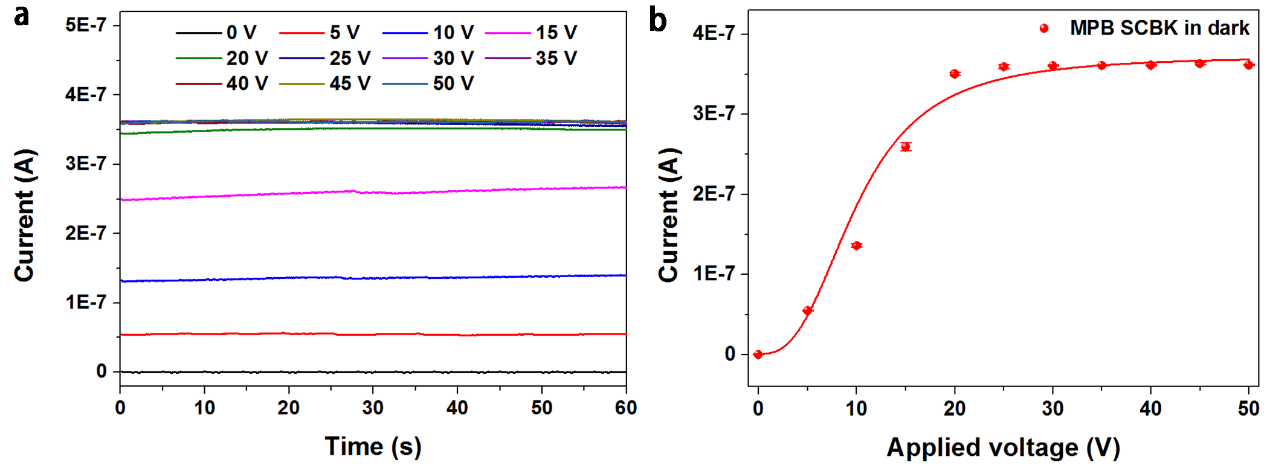


**Figure S3**. **Scanning electron microscope (SEM) image and Energy Dispersive Spectrometry (EDS) mapping of MPB SCBK.** **a** SEM image of MPB SCBK. **b-f** EDS mapping of the MPB SCBK with uniform elemental distribution of gold, bromide, lead, carbon, and nitrogen, respectively. **g** The EDS spectrum in selected area.


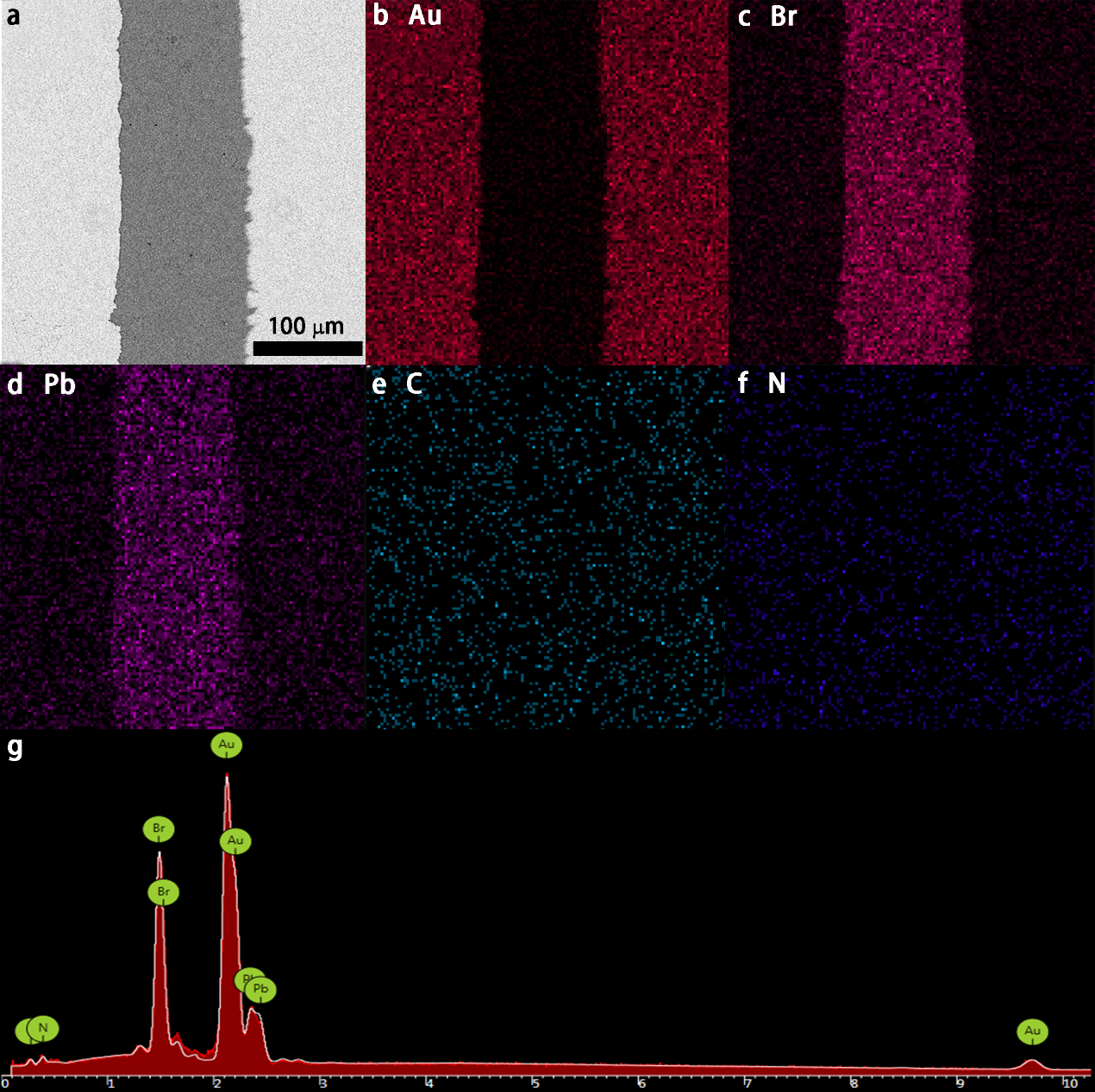


**Figure S4**. **a** Optical image, **b** three-dimensional (3D) pseudo color plots and **c** thickness information of Au electrode deposited on MPB SCBK.


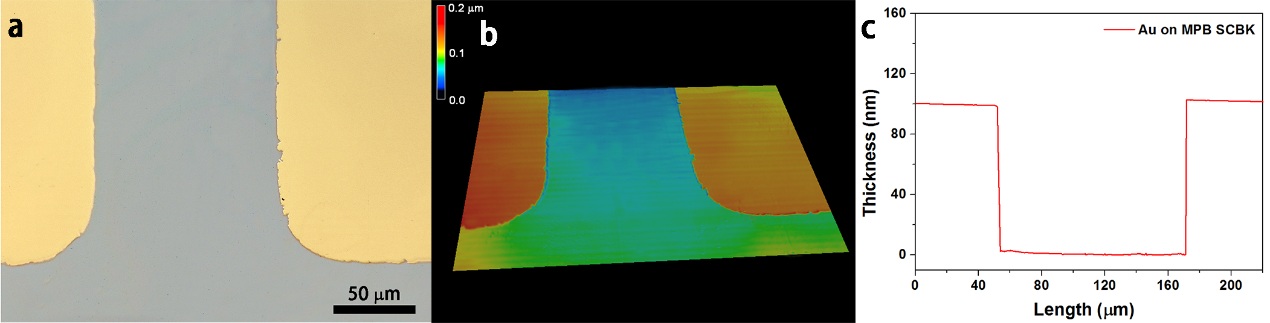



**Figure S5**. **The relationship between electrical poling intensity and applied voltage with electrode spacing of 120 μm.**

**Figure S6**. **TRPL of MPB SCBK around cathode with different applied bias.**


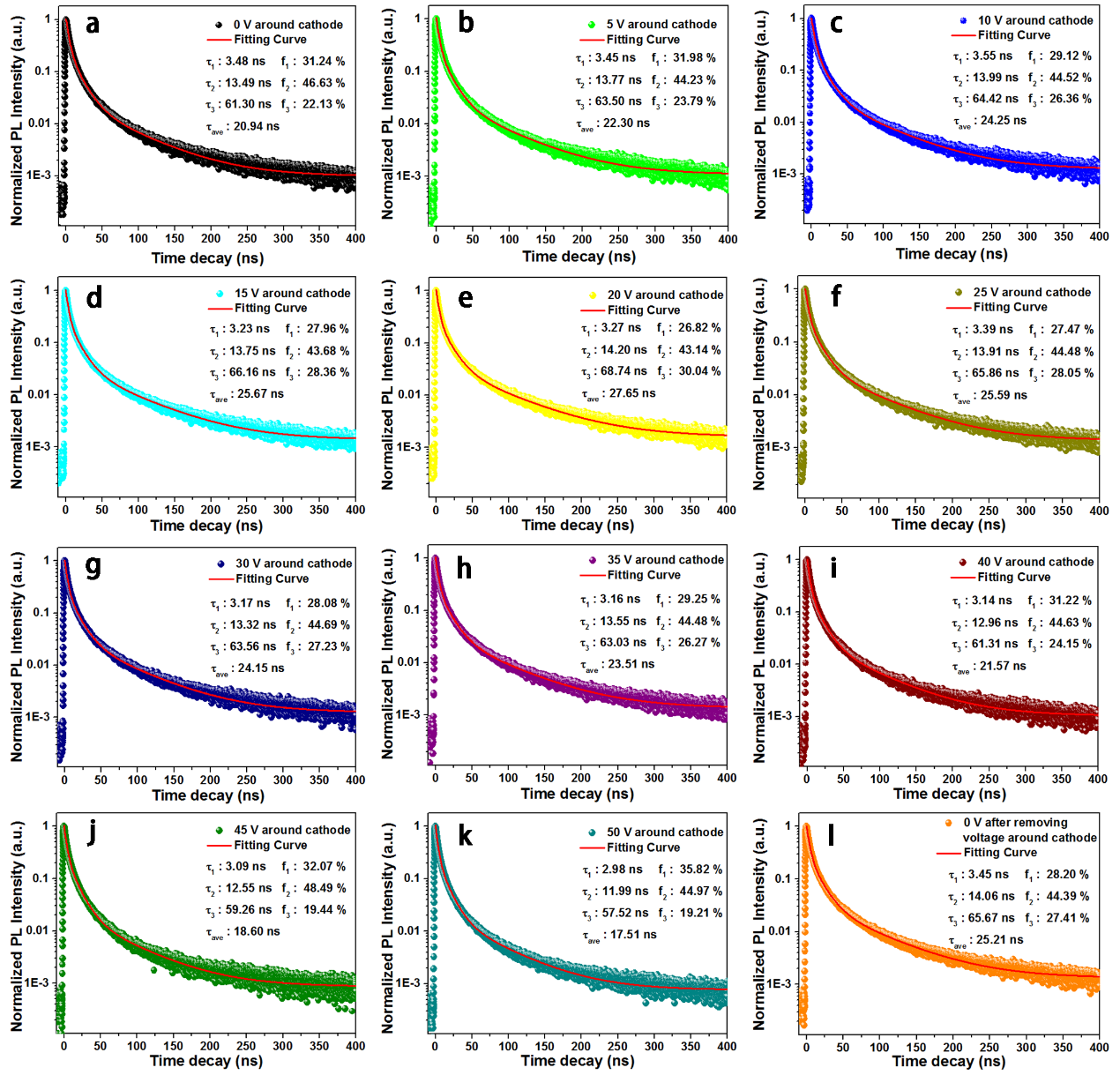


**Figure S7**. **TRPL of MPB SCBK in center region with different applied bias.**


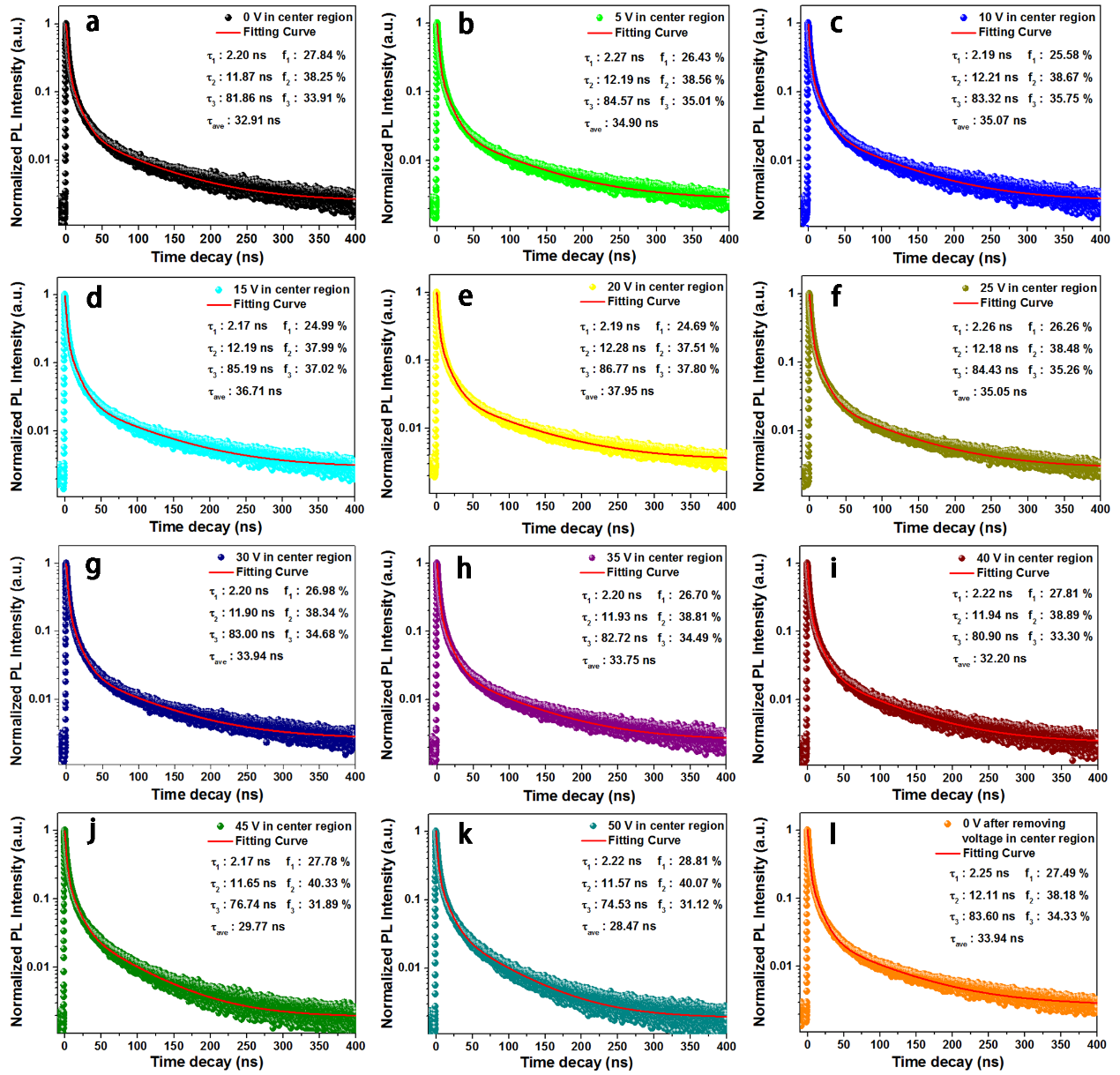


**Figure S8**. **TRPL of MPB SCBK around anode with different applied bias.**


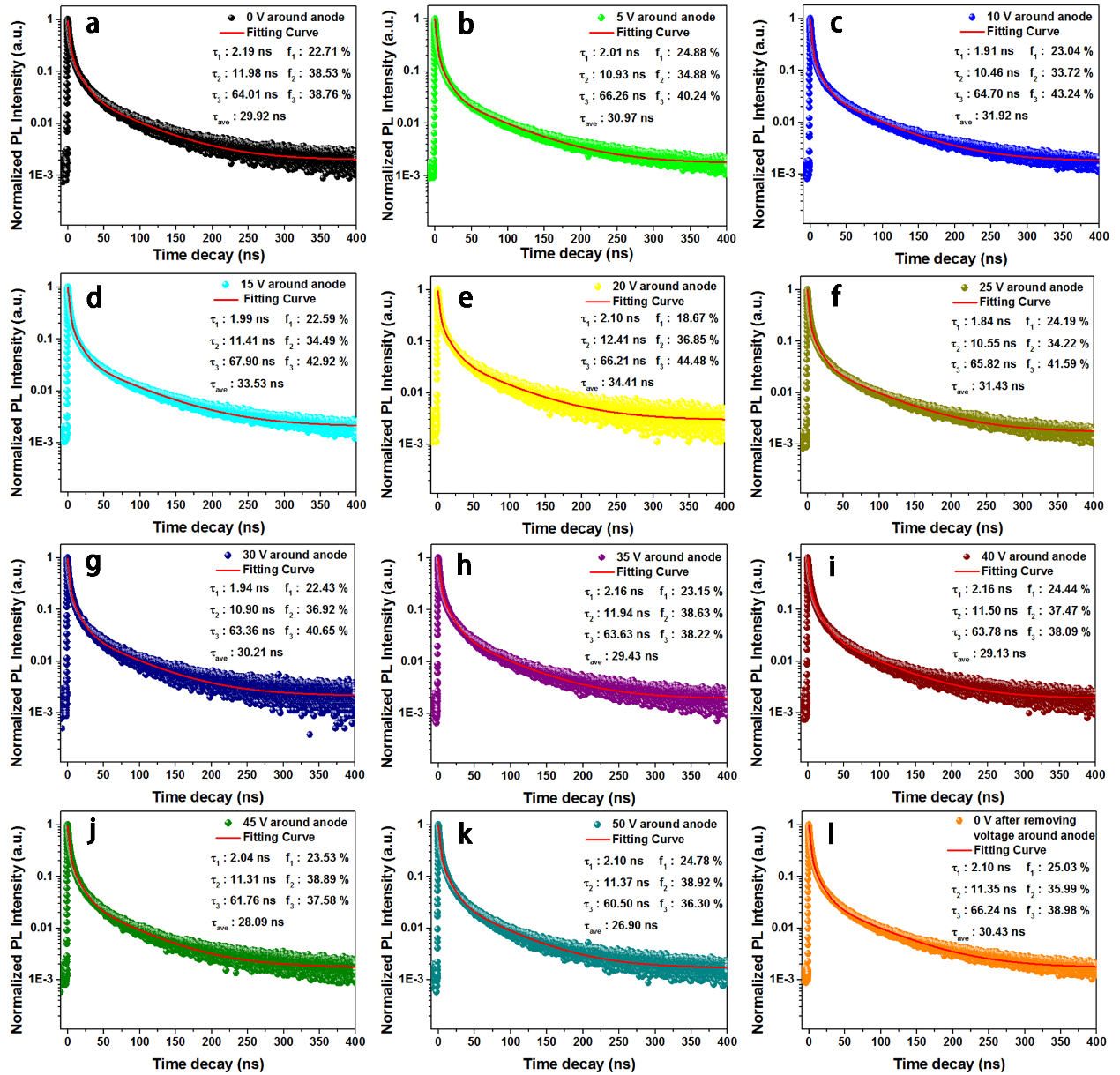


**Table** **S1** TRPL information of MPB SCBK around cathode with different applied bias under 532 nm laser.

| **Applied bias** | **τ_1_ (ns)** | **f_1_ (%)** | **τ_2_ (ns)** | **f_2_ (%)** | **τ_3_ (ns)** | **f_3_ (%)** | **τ_ave_ (ns)** |
| --- | --- | --- | --- | --- | --- | --- | --- |
| **0 V** | 3.48 | 31.24 | 13.49 | 46.63 | 61.30 | 22.13 | 20.94 |
| **5 V** | 3.45 | 31.98 | 13.77 | 44.23 | 63.50 | 23.79 | 22.30 |
| **10 V** | 3.55 | 29.12 | 13.99 | 44.52 | 64.42 | 26.36 | 24.25 |
| **15 V** | 3.23 | 27.96 | 13.75 | 43.68 | 66.16 | 28.36 | 25.67 |
| **20 V** | 3.27 | 26.82 | 14.20 | 43.14 | 68.74 | 30.04 | 27.65 |
| **25 V** | 3.39 | 27.47 | 13.91 | 44.48 | 65.86 | 28.05 | 25.59 |
| **30 V** | 3.17 | 28.08 | 13.32 | 44.69 | 63.56 | 27.23 | 24.15 |
| **35 V** | 3.16 | 29.25 | 13.55 | 44.48 | 63.03 | 26.27 | 23.51 |
| **40 V** | 3.14 | 31.22 | 12.96 | 44.63 | 61.31 | 24.15 | 21.57 |
| **45 V** | 3.09 | 32.07 | 12.55 | 48.49 | 59.26 | 19.44 | 18.60 |
| **50 V** | 2.98 | 35.82 | 11.99 | 44.97 | 57.52 | 19.21 | 17.51 |
| **0 V after removing bias** | 3.45 | 28.20 | 14.06 | 44.39 | 65.67 | 27.41 | 25.21 |

**Table** **S2** TRPL information of MPB SCBK in center region with different applied bias under 532 nm laser.

| **Applied bias** | **τ_1_ (ns)** | **f_1_ (%)** | **τ_2_ (ns)** | **f_2_ (%)** | **τ_3_ (ns)** | **f_3_ (%)** | **τ_ave_ (ns)** |
| --- | --- | --- | --- | --- | --- | --- | --- |
| **0 V** | 2.20 | 27.84 | 11.87 | 38.25 | 81.86 | 33.91 | 32.91 |
| **5 V** | 2.27 | 26.43 | 12.19 | 38.56 | 84.57 | 35.01 | 34.90 |
| **10 V** | 2.19 | 25.58 | 12.21 | 38.67 | 83.32 | 35.75 | 35.07 |
| **15 V** | 2.17 | 24.99 | 12.19 | 37.99 | 85.19 | 37.02 | 36.71 |
| **20 V** | 2.19 | 24.69 | 12.28 | 37.51 | 86.77 | 37.80 | 37.95 |
| **25 V** | 2.26 | 26.26 | 12.18 | 38.48 | 84.43 | 35.26 | 35.05 |
| **30 V** | 2.20 | 26.98 | 11.90 | 38.34 | 83.00 | 34.68 | 33.94 |
| **35 V** | 2.20 | 26.70 | 11.93 | 38.81 | 82.72 | 34.49 | 33.75 |
| **40 V** | 2.22 | 27.81 | 11.94 | 38.89 | 80.90 | 33.30 | 32.20 |
| **45 V** | 2.17 | 27.78 | 11.65 | 40.33 | 76.74 | 31.89 | 29.77 |
| **50 V** | 2.22 | 28.81 | 11.57 | 40.07 | 74.53 | 31.12 | 28.47 |
| **0 V after removing bias** | 2.25 | 27.49 | 12.11 | 38.18 | 83.60 | 34.33 | 33.94 |

**Table** **S3** TRPL information of MPB SCBK around anode with different applied bias under 532 nm laser.

| **Applied bias** | **τ_1_ (ns)** | **f_1_ (%)** | **τ_2_ (ns)** | **f_2_ (%)** | **τ_3_ (ns)** | **f_3_ (%)** | **τ_ave_ (ns)** |
| --- | --- | --- | --- | --- | --- | --- | --- |
| **0 V** | 2.19 | 22.71 | 11.98 | 38.53 | 64.01 | 38.76 | 29.92 |
| **5 V** | 2.01 | 24.88 | 10.93 | 34.88 | 66.26 | 40.24 | 30.97 |
| **10 V** | 1.81 | 23.04 | 10.46 | 33.72 | 64.70 | 43.24 | 31.92 |
| **15 V** | 1.99 | 22.59 | 11.41 | 34.49 | 67.90 | 42.92 | 33.53 |
| **20 V** | 2.10 | 18.67 | 12.41 | 36.85 | 66.21 | 44.48 | 34.41 |
| **25 V** | 1.84 | 24.19 | 10.55 | 34.22 | 65.82 | 41.59 | 31.43 |
| **30 V** | 1.94 | 22.43 | 10.90 | 36.92 | 63.36 | 40.65 | 30.21 |
| **35 V** | 2.16 | 23.15 | 11.94 | 38.63 | 63.63 | 38.22 | 29.43 |
| **40 V** | 2.16 | 24.44 | 11.50 | 37.47 | 63.78 | 38.09 | 29.13 |
| **45 V** | 2.04 | 23.53 | 11.31 | 38.89 | 61.76 | 37.58 | 28.09 |
| **50 V** | 2.10 | 24.78 | 11.37 | 38.92 | 60.50 | 36.30 | 26.90 |
| **0 V after removing bias** | 2.10 | 25.03 | 11.35 | 35.99 | 66.24 | 38.98 | 30.43 |

**The simplified derivation process of equation 1**

Using single-photon excitation (in our experiment), the effect of surface states in the single crystals can be quantified by extracting the surface recombination velocity (*S*) using the following equation^1^

$$\frac{1}{\tau_{S}}=\frac{2\alpha^{2}D}{1+\sqrt{1+\frac{8\alpha^{2}D^{2}}{S^{2}}}}$$

where *α* is the absorption coefficient at the excitation wavelength (at 532 nm about 70862 cm^-1^)^2^, and *D=μkT/q* is the carrier diffusion coefficient. This expression is applicable if the sample thickness is much larger than the optical penetration depth (*α*^-1^), which is the case for the single-photon excitation study on our crystal.

Due to $\frac{8\alpha^{2}D^{2}}{S^{2}}\gg1$, the above expression can be simplified as follows to get the equation 1 in our manuscript:

$$\frac{1}{\tau_{S}}=\frac{2\alpha^{2}D}{1+\sqrt{1+\frac{8\alpha^{2}D^{2}}{S^{2}}}}\approx\frac{2\alpha^{2}D}{\sqrt{8\alpha^{2}D^{2}/S^{2}}}=\frac{2\alpha^{2}D}{2\sqrt{2}\alpha D/S}=\frac{\alpha S}{\sqrt{2}}$$

**Figure S9**. **Steady-state PL of MPB SCBK with delay time under no bias.**


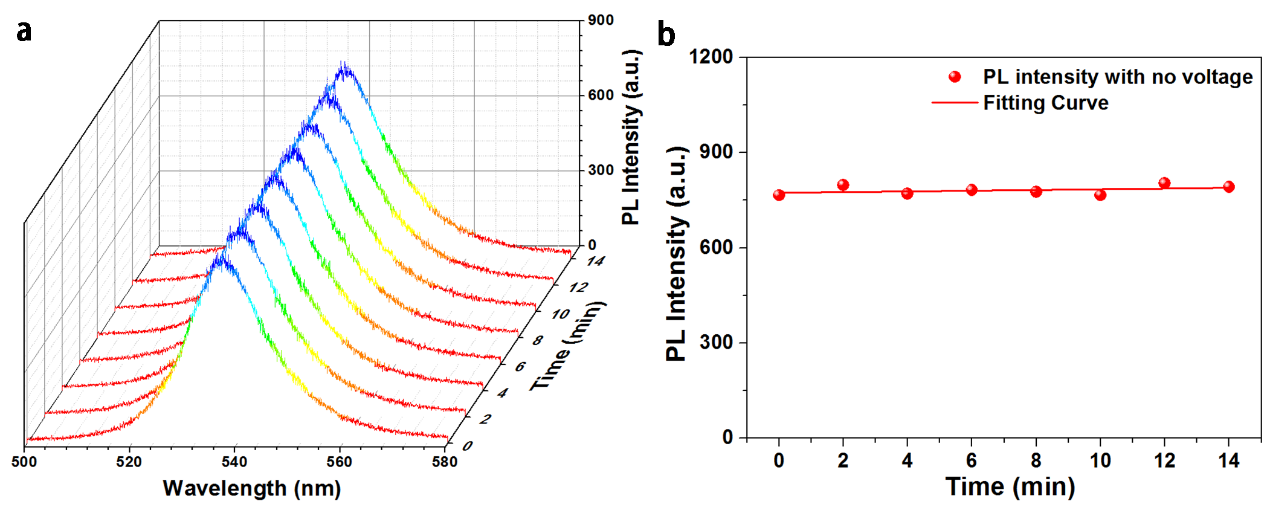


**Table** **S4** Lifetime, surface recombination velocity, surface defects density and PL intensity information of MPB SCBK in different postion before and after voltage regulated.

|  | **Position** | **Voltage (V)** | **τ_ave_ (ns)** | **S (cm s^-1^)** | **N_t_ (cm^-2^)** | **PL (a.u.)** |
| --- | --- | --- | --- | --- | --- | --- |
| **Before voltage regulated** | Around cathode | 0 | 20.94 | 950 | 2.57×10^10^ | 322 |
|  | Center region | 0 | 32.91 | 604 | 1.63×10^10^ | 629 |
|  | Around anode | 0 | 29.92 | 665 | 1.79×10^10^ | 625 |
| **After voltage regulated** | Around cathode | 0 | 25.21 | 789 | 2.13×10^10^ | 452 |
|  | Center region | 0 | 33.94 | 586 | 1.58×10^10^ | 722 |
|  | Around anode | 0 | 30.43 | 653 | 1.76×10^10^ | 1001 |

**Figure S10**. **Typical *J-V* hysteresis loops over 300 cycles of MPB SCBK in dark with different bias polarizing for 1 min in air at room temperature.** The voltage sweep sequence is 0 V → 25 V → 0 V → -25 V→ 0 V.


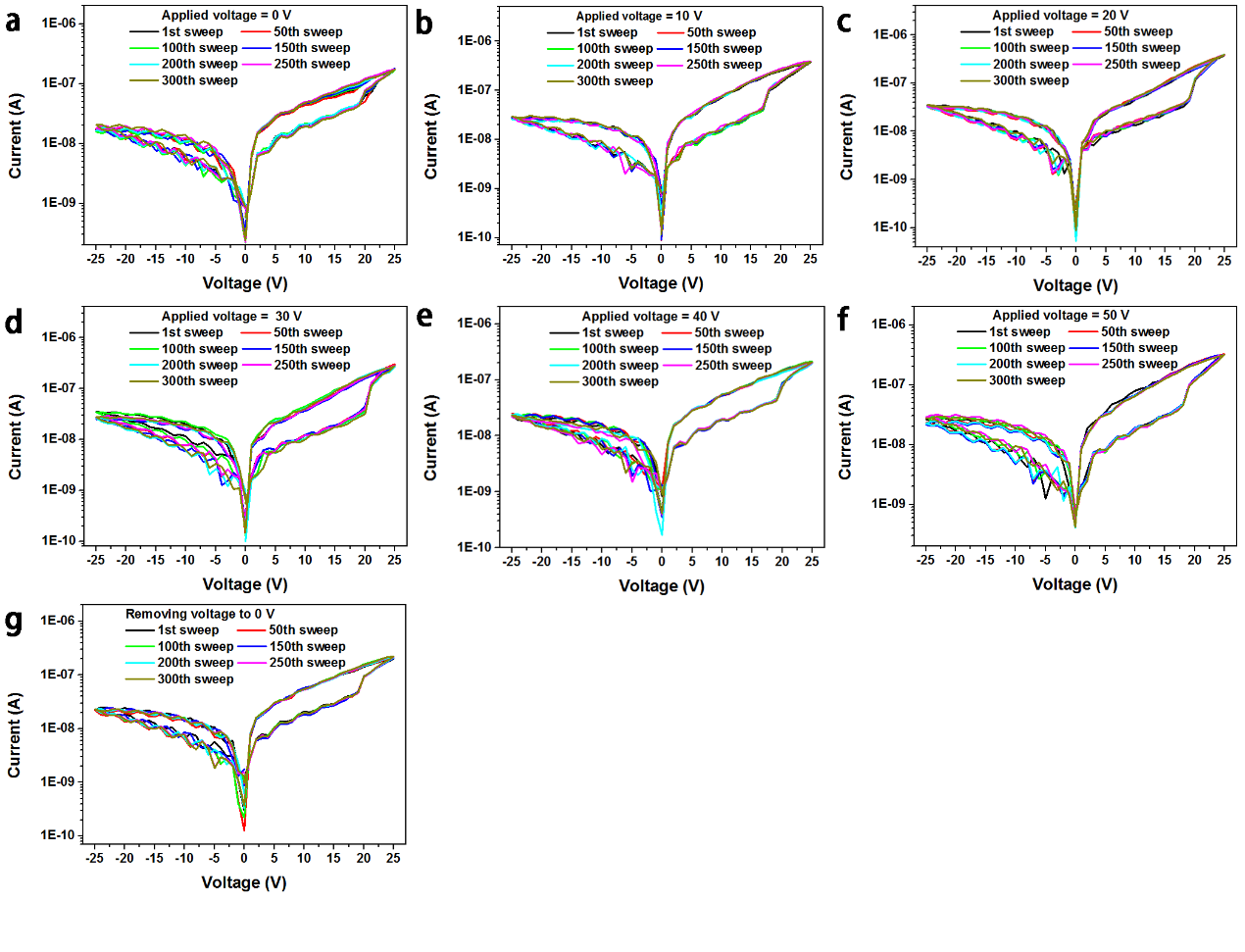


**Figure S11**. **Multi resistance states (HRS and LRS) of MPB SCBK in dark with different bias polarizing for 1 min at V_readout_= 1 V.**


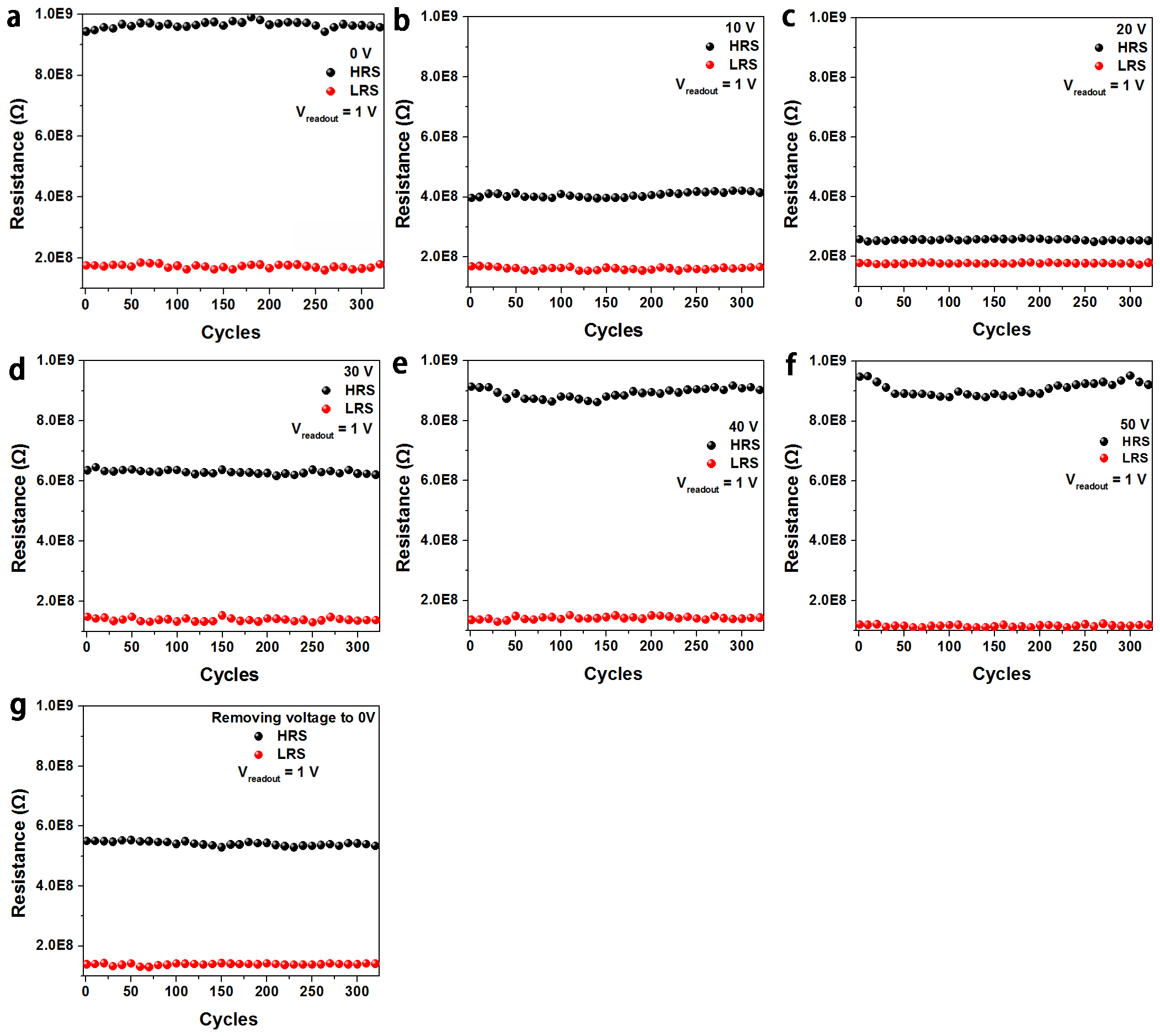


**Table** **S5** HRS, LRS, HRS/LRS ratio of MPB SCBK before and after voltage regulated at V_readout_= 1 V.

| **Voltage regulated** | **HRS (Ω)** | **LRS (Ω)** | **HRS/LRS ratio** |
| --- | --- | --- | --- |
| **Before** | 9.65$\times$10^8^ | 1.72$\times$10^7^ | 5.62 |
| **After** | 5.41$\times$10^8^ | 1.38$\times$10^7^ | 3.91 |

**References**

1. Wang, H. et al. One- and two-photon-excited time-resolved photoluminescence investigations of bulk and surface recombination dynamics in ZnSe. *Journal of Applied Physics* **83**, 4773-4776 (1998).

2. Yang, Y. et al. Low surface recombination velocity in solution-grown CH_3_NH_3_PbBr_3_ perovskite single crystal. *Nature Communication* **6**, 7961 (2015).
